# Micheliolide Derivative DMAMCL Inhibits Glioma Cell Growth In Vitro and In Vivo

**DOI:** 10.1371/journal.pone.0116202

**Published:** 2015-02-06

**Authors:** Yinghong An, Wanjun Guo, Linna Li, Chengwang Xu, Dexuan Yang, Shanshan Wang, Yaxin Lu, Quan Zhang, Jiadai Zhai, Hongxia Fan, Chuanjiang Qiu, Jie Qi, Yue Chen, Shoujun Yuan

**Affiliations:** 1 Clinical Laboratory Center, Chinese PLA Air Force General Hospital, Haidian, Beijing 100142, PR China; 2 Department of Pharmacology and Toxicology, Beijing Institute of Radiation Medicine, Beijing 100850, PR China; 3 College of Pharmacy, The State Key Laboratory of Elemento-Organic Chemistry, Collaborative Innovation Center of Chemical Science and Engineering (Tianjin), and Tianjin Key Laboratory of Molecular Drug Research, Nankai University, Tianjin 300071, PR China; 4 Accendatech Co., Ltd., Tianjin 300384, PR China; Complutense University, SPAIN

## Abstract

**Background:**

There is no highly effective chemotherapy for malignant gliomas to date. We found that dimethylaminomicheliolide (DMAMCL), a selective inhibitor of acute myeloid leukemia (AML) stem/progenitor cells, inhibited the growth of glioma cells.

**Methods:**

The distribution of DMAMCL in brain was analyzed by an ultraperformance liquid chromatography-mass spectrometry (UPLC-MS/MS) system. The anti-tumor evaluations of DMAMCL in vitro were performed by MTT, FACS and RT-PCR. In vivo, the mixture of C6 cells and matrigel was injected into caudatum, and the anti-tumor activity of DMAMCL was evaluated by tumor growth and rat survival. The toxicity of DMAMCL was evaluated by body weight, daily food intake, hematological or serum biochemical analyses, and histological appearance of tissues.

**Results:**

The IC_50_ values of DMAMCL against the C6 and U-87MG cell lines in vitro were 27.18 ± 1.89 μM and 20.58 ± 1.61 μM, respectively. DAMMCL down-regulated the anti-apoptosis gene Bcl-2 and increased apoptosis in C6 and U-87MG cells in a dose-dependent manner. In a C6 rat tumor model, daily administration of DMAMCL for 21 days reduced the burden of C6 tumors by 60% to 88% compared to controls, and more than doubled the mean lifespan of tumor-bearing rats. Distribution analysis showed that the DMAMCL concentration was higher in the brain than in plasma. Evaluations for toxicity revealed that oral administration of DMAMCL at 200 or 300 mg/kg once a day for 21 days did not result in toxicity.

**Conclusions:**

These results suggest that DMAMCL is highly promising for the treatment of glioma.

## Introduction

Malignant glioma (MG) is a highly invasive, heterogeneous cancer that arises from glial cells in the nervous system. It is the most fatal primary brain cancer [1, 2]. Despite enormous research efforts, there is currently no cure for MG, and only very limited progress has been made to control the course of disease. The average survival time of patients with MG is only 7.0 to 14.6 months with treatment [2–5].

In both low- and high-grade MG, microscopic infiltrations into normal brain tissue [6] represent the main reason for failed surgical resection. The treatments of MG remain largely palliative. Currently, the standard therapeutic approach is adjuvant radiotherapy and chemotherapy after surgery. Although the addition of radiotherapy and chemotherapy prolongs patient survival [5, 7], they has harmful side effects, such as loss of cognitive function, inflammatory responses to chemotherapy, or secondary cancer from radiotherapy [8]. Temozolomide (TMZ), an oral drug that alters DNA in tumor cells to induce tumor cell death, is part of a standard postoperative treatment regimen for MG [9]. Adjuvant therapy with TMZ has shown promising results in terms of increasing patient survival [5], however, its use is hindered by major side effects, such as lymphopenia and abnormally low levels of leukocytes [10]. Another anticancer agent, bevacizumab, has earned FDA approval to treat brain tumors. However, it inhibits tumor blood vessel formation rather than directly acts upon tumor cells. It remains unclear whether this therapy will advance treatment significantly. On the other hand, the passage of chemotherapy drugs across vascular endothelial cells greatly affects the speed and efficiency of therapeutic action [11]. The ability to cross these cells is determined by the hydrophobicity of the chemotherapeutic agent. The blood brain barrier (BBB) has active efflux transporters to prevent systemically administered drugs from entering the brain, hampering both conventional and targeted therapies [12, 13]. However, it is essential for therapeutics to cross the BBB. Novel compounds that can safely and effectively treat MG are urgently needed not only for therapeutic purposes, but also for decreasing economic burden.

Parthenolide (PTL) is a sesquiterpene lactone (SL) isolated from Tanacetum parthenium (Feverfew) that was reported to inhibit the growth of glioblastoma cell line U-87 MG [14]. The maximum response was achieved at 20 μM, resulting in a 30% to 40% decrease in cell viability [14]. However, PTL is unstable under both acidic and basic conditions [15]. The compound micheliolide (MCL) is a guaianolide sesquiterpene lactone (GSL). It has been proved that MCL is more stable than PTL[16]. MCL greatly reduced the number of colony-forming units of primary acute myeloid leukemia (AML) cells [16]. The affordable (about 5$/g) dimethylaminomicheliolide (DMAMCL) is a chemotherapeutic agent that is synthesized through a Michael addition of MCL. DMAMCL slowly releases MCL as a metabolite in plasma. This drug has shown superior therapeutic efficacy in AML models [16].

In this study, we investigated the biodistribution of DMAMCL, its potential to inhibit the growth of MG, and its probable mechanisms in vivo and in vitro. The results indicate that the concentration of DMAMCL in brain is remarkably higher than that in plasma, and that DMAMCL exhibits significant anti-MG activities in vitro or vivo.

## Materials and Methods

### Ethics and statement

Experiments were designed and performed in accordance with the standards of the Guide for the Care and Use of Laboratory Animals as adopted by the Animal Experimentation Ethics Committee of the Military Medical Science Academy of the People’s Liberation Army in China. We confirm that this research has been specifically approved by this committee and the certification numbers are respectively SCXK (jing) 2005–0013 and SCXK (army) 2012–001 for two groups of Wistar rats. We have done our best to report animal studies following the ARRIVE guidelines[17]. And all efforts were taken to minimize the suffering and stress of animals.

### DMAMCL synthesis

**Fig 1:**
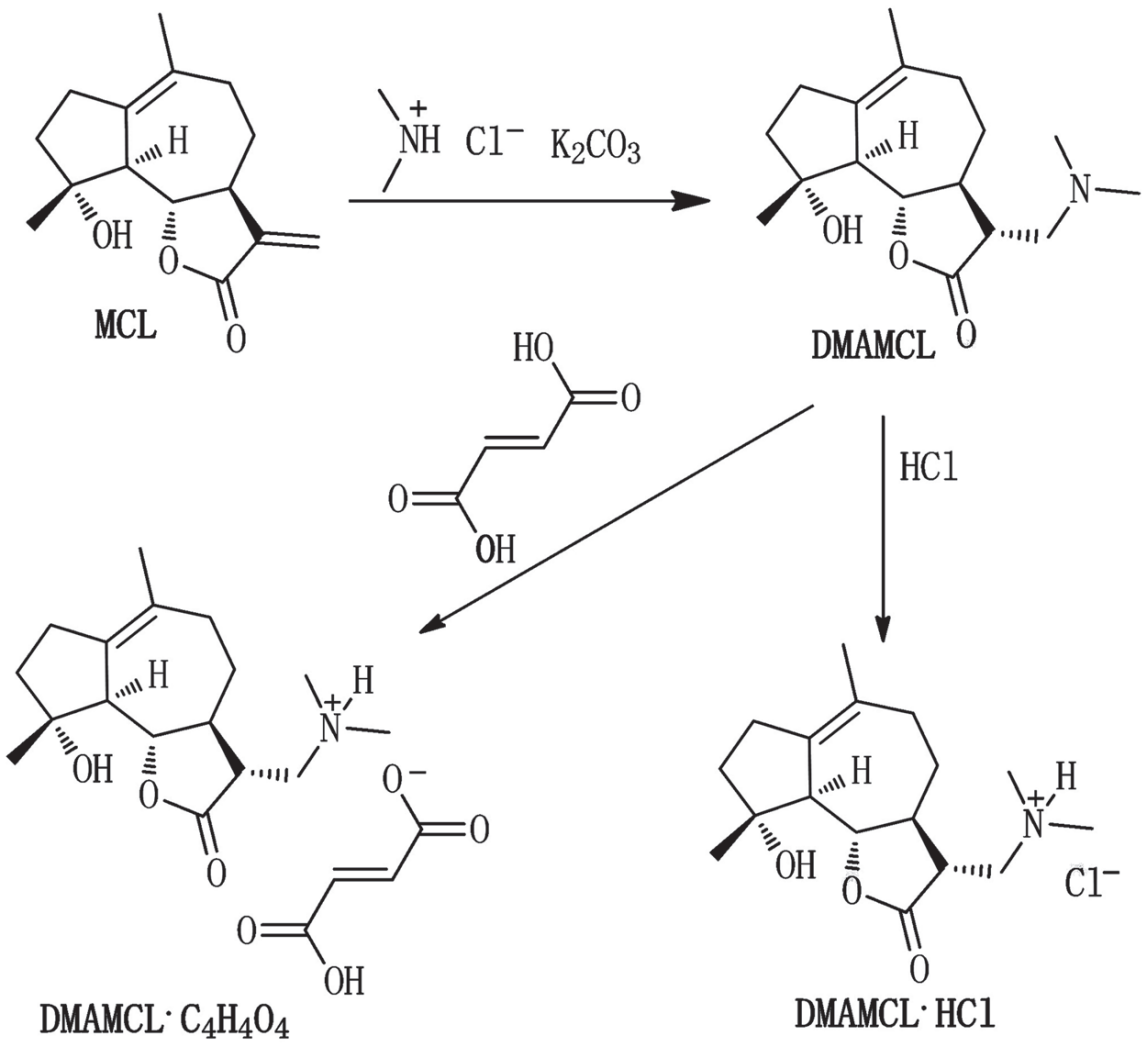
Molecular structures of MCL, DMAMCL, and DMAMCL salts.

DMAMCL was generously provided by Accendatech Co., Ltd. (Tianjin, China). Briefly, MCL was prepared as previously described [18]. DMAMCL was obtained as a white powder by adding dimethylamine to MCL (Fig. 1) and the molecular weight is 409.21 [19]. The addition of 1 eq HCl yielded DMAMCL•HCl and was followed by the addition of 1 eq fumarate to yield DMAMCL•C_4_H_4_O_4_ [16, 19]. In this study, DMAMCL•C_4_H_4_O_4_ (short for DMAMCL) was used, which was purified and certified as conventional procedures [19]. Briefly, Me_2_NH•HCl (1.5 g, 18 mmol), K_2_CO_3_ (5.0 g, 36 mmol), and CH_2_Cl_2_ (100 ml) was mixed and stirred at room temperature until all the materials were dissolved. Then the resulting solution was treated with MCL (300 mg, 1.2 mmol) and the reaction mixture was stirred at room temperature for 3 hr again. The reaction mixture was subjected to concentration and re-dissolved in CH_2_Cl_2_. The resulting organic solution was washed with water, and then the organic layer was dried with Na_2_SO_4_. Dimethylaminomicheliolide was obtained by concentrating the above mother liquid. Subsequently, the crude dimethylaminomicheliolide (300 mg) was dissolved in CH_2_Cl_2_ (5 ml) again and treated with fumarate (0.1 N) until the pH value of the aqueous phase was 4. The aqueous phase was extracted with CH_2_Cl_2_ (10 ml) and lyophilized to afford compound DMAMCL•C_4_H_4_O_4_. All the obtained compounds were certified by an Agilent 6520 Q-TOF LC/MS instrument.

### Pharmacokinetic studies in rats

Adult Wistar rats (200–250 g) were obtained from the Animal Center at the Academy of Military Medical Sciences and had animal certification of SCXK (jing) 2005–0013. Animals were housed in collective cages at a temperature of 23 ± 2°C and relative humidity of 40% to 70% under a 12h light-dark cycle. All rats had free access to food and water. Before drug administration, rats were fasted for 12 h and water was freely available.

Aqueous DMAMCL solutions were administered orally (100 mg/kg) (n = 6 rats per group). Blood and tissue samples from rats were collected at the setting time and analyzed on an ultraperformance liquid chromatography-mass spectrometry (UPLC-MS/MS) system (Waters/Micromass, Milford, MA) using noncompartmental analyses. All the biological specimens were detected at once or stored at -80°C for detection.

### Cell lines and cell culture

C6 and U-87MG cell lines were obtained from the American Type Culture Collection (ATCC) and cultured in our laboratory. Rat C6 glioma cells were cultured in F12K nutrient medium (Gibco, Grand Island, UK) supplemented with 15% horse serum (Auckland, NZ), 2.5% fetal bovine serum (FBS), 100 U/mL penicillin, and 100 mg/mL streptomycin (Gibco, Paisley, Scotland, UK). Human U-87MG cells were cultured in minimum essential medium (MEM) supplemented with 10% FBS, 1% nonessential amino acids, 100 U/mL penicillin, and 100 mg/mL streptomycin. Cells were cultured under a humidified atmosphere containing 5% CO_2_ at 37°C, and medium was changed every 1 to 2 days.

### Cell proliferation assays

Tumor cells were seeded in 96-well plates at a density of 3500 cells/well. After 24 h in culture, cells were exposed to different concentrations of DMAMCL or medium alone (control) for 72 h. Cell viability was determined by MTT assays. Briefly, MTT regent (0.5 mg/mL) was added to wells, and cells were incubated for 4 h at 37°C. After removing the supernatant, 200 μL of dimethyl sulfoxide (DMSO) (Fluka, Switzerland) was added to dissolve formazan crystals. The absorbance at 570 nm was measured by a microplate reader (Thermo Labsystems, Helsinki, Finland). Control absorbance was taken as 100%. The absorbance of DMAMCL-treated cells was taken as percentage survival. The 50% inhibitory concentration (IC_50_) was determined by non-linear regression analyses of the dose-response curves (Microcal Origin 7.0 software).

### Apoptosis measurements by flow cytometry

Glioma cells (10^4^) in the logarithmic growth phase were seeded in 6-well plates for 24 h. Cells were treated with DMAMCL at 240, 120, 60, 30, 15, or 0 μM. They were removed from culture plates with 0.5% trypsin containing ethylene diamine tetraacetic acid (EDTA). Apoptosis of C6 cells was measured using an Annexin V and propidium iodide (PI) apoptosis detection kit (Invitrogen, USA) and a Beckman flow cytometer (Cytomics FC500, Beckman Coulter, USA).

### Western blotting analysis

The cellular proteins were extracted from 10^7^ cultured cells after drug treatments using RIPA lysis buffer (Promega, USA). Western blotting analysis was performed to determine cellular responses targeting apoptosis-related proteins including Bcl-2 and Bax. Briefly, C6 and U-87MG cells were treated with different doses of DMAMCL for 24 hr at 37°C. Then cells were washed twice with cold phosphate buffered saline (PBS) and lysed in RIPA buffer containing 1mmol/l PMSF (Pheylmethylsulfonyl fluoride, Sigma, USA) on ice for 30 min. The total proteins of cells were obtained by centrifugation at 12000 rpm for 15 min. Next, the lysate proteins were resolved by SDS-PAGE and transferred to the Immobilon-P membranes (Millipore, Billerica, MA, USA). The membranes were blocked by 5% nonfat milk in Tris-buffered saline with 0.1% Tween 20 (TBST) and immunoblotted with the antibodies against β-actin, Bcl-2 and Bax (Cellsignaling Technology, USA) at 4°C overnight. Then membranes were incubated with HRP-conjugated secondary antibody for 1.5 hr at room temperature. The targeting antibodies were stained with enhanced chemiluminescent reagents (Millipore, Billerica, MA, USA) and visualized by ChemiScope 3600 Mini systerm (Clinx Science Instruments, China).

### Intracerebral tumor implantation and anti-tumor activity of DMAMCL in vivo

Adult Wistar rats (120–180 g, age 4–8 weeks) were obtained from the Animal Center at the Academy of Military Medical Sciences and had animal certification of SCXK (army) 2012–001. Animals were housed in collective cages at a temperature of 23 ± 2°C and relative humidity of 40% to 70%. All rats had free access to food and water.

Intracerebral tumor implantations were performed according to the following procedures. C6 tumors were maintained as subcutaneous tumors in rats. Subcutaneous masses were excised, dissociated, and mixed with matrigel on ice (BD Biosciences) at a ratio of 1:1. After removing of the hair on the skull, a small midline incision in the scalp was gently made under anesthesia with 2% sodium pentobarbital at a dose of 40mg/kg. C6 cells in matrigel (0.1 mL of the cell/matrigel mixture containing 10^8^ cells /mL) were carefully injected into the right frontal cortex by a special 16-gauge stainless steel needle with 5 mm needle tubing. Implantation coordinates were determined from Swanson’s Stereotaxic Atlas guidelines (1992): 1.0 mm anterior and 3.0 mm lateral to bregma, at a depth of 5.0 mm from the brain surface [20]. Employing this approach largely avoided intraventricular injection of cells, subsequent spinal dissemination, or extracranial tumor development. The syringe was slowly retracted, the hole was sealed with bone wax, and the incision was sutured under aseptic conditions. The surgical process was limited in 5 minutes in order to minimize the suffering and stress of rats and mortality from the procedure was < 2%. Then 0.015 mg/kg buprenorphine (s.c.) was administered to relieve the pain. The rats were replaced in cages and allowed to recover from anesthesia.

Rats were divided into the following 5 treatment groups 24 h after surgery (n = 9 rats /group) according to rat weight: 1) vehicle (normal saline: NS); 2) vincristine (VCR) at 100μg/kg; 3) DMAMCL at 25 mg/kg; 4) DMAMCL at 50 mg/kg; and 5) DMAMCL at 100 mg/kg. All drugs were prepared in NS and administered once daily for 21 days. DMAMCL was administered orally, and VCR was injected into the caudal vein. Rats were weighed and monitored for signs of mortality daily. Twenty one days into the treatment, rats were euthanized via CO2 asphyxiation under deep anesthesia by sodium pentobarbital. Brains and tumors were separated from the cerebellum and medulla oblongata, and weighed, photographed, fixed in formalin, and embedded in paraffin for sectioning. The tumor: brain weight ratio was assessed to observe the degree of tumor cell growth in brain. Serial sections (5 μm in thickness) were stained by hematoxylin and eosin (HE).

The survival time analyses were performed in C6 intracerebral glioma rats. Rats were divided into the following 5 treatment groups 24 h after surgery (n = 10 rats /group) according to rat weight as mentioned above: 1) vehicle; 2) VCR; 3) DMAMCL at 25 mg/kg; 4) DMAMCL at 50 mg/kg; and 5) DMAMCL at 100 mg/kg. Animals were monitored daily for signs of mortality and weight. Rats were sacrificed when a loss of mobility, lethargy, hemorrhaging around the eyes, or a hunched posture was observed as previously described [21]. The observation time was terminated when all the vehicle rats died. Then the other rats were euthanized by CO2 asphyxiation.

### Toxicity of DMAMCL

Wistar rats (150–170 g) were divided into the following 3 groups (6 rats /group): 1) vehicle control, 2) DMAMCL at 200 mg/kg, and 3) DMAMCL at 300 mg/kg. DMAMCL was administered orally once a day for 21 days. Rats were weighed on days 0, 3, 7, 11, 14, 17, and 21. Total food intake was measured as the weight of food consumed during a 24-h period on days 3, 8, 15, and 22. On the 23rd day, rats were anesthetized and sacrificed via CO2 asphyxiation. Peripheral blood was collected for hematological and serum biochemical analyses using a blood cell counter (Medonic M16, Boule medical AB, Sweden) and a Hitachi 7180 Automatic Analyzer (Japan). Tissues samples from the liver, spleen, lung, kidney, and brain were collected and embedded in paraffin for H&E staining.

Locomotor activity was used to assess neurological effects of DMAMCL. Wistar rats (150–170 g) were divided into the following 3 groups (10 rats /group): 1) vehicle control, 2) DMAMCL at 100 mg/kg, and 3) DMAMCL at 300 mg/kg. DMAMCL was administered orally once a day for 21 days. Rat locomotor activities were monitored on days 1, 10, 21 and 28 using Aninal Behavioral Analysis System (AniLab Software and Instruments Co, Ltd, China). Briefly, rats were gently placed in the locomotor activity boxes equipped with infrared photobeams and individual total distance (cm) of rat running was recorded during a test period of 60 min. The test apparatus was cleaned to eliminate odors for the next test after each animal was tested.

### Statistical analyses

Data were expressed as the mean ± SEM from at least 3 independent experiments. One-way ANOVAs followed by Tukey’s post-hoc tests were performed to analyze statistical differences between different treatment groups. A p value < 0.05 was considered statistically significant.

## Results

### Biodistribution of DMAMCL

**Fig 2:**
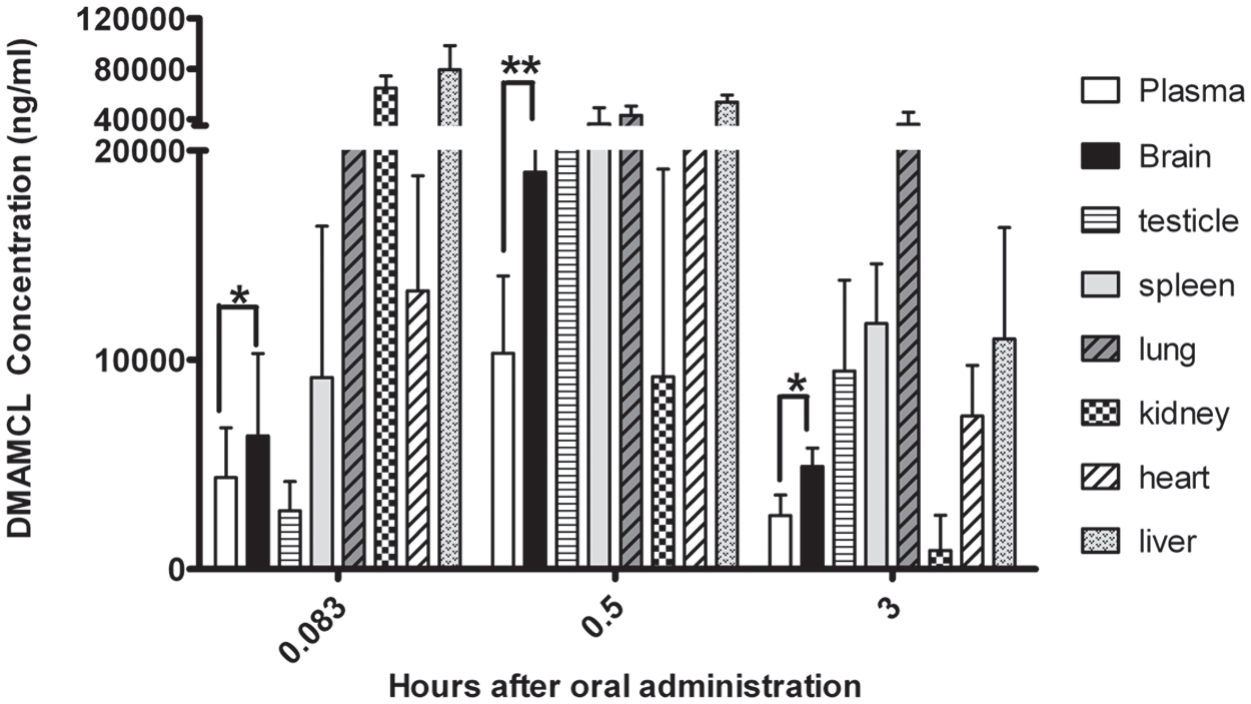
Biodistribution of DMAMCL administered orally. DMAMCL concentrations over time in rats after a single oral dose (100 mg/kg) (n = 6) in plasma, brain, spleen, lung, kidney, heart and liver were detected by UPLC-MS/MS. Statistical significance between plasma and brain is indicated by asterisks (*p < 0.01 compared to vehicle; **p < 0.01 compared to vehicle).

To measure the biodistribution of DMAMCL in rats, a single oral dose of an aqueous DMAMCL solution (100 mg/kg) was given, and the concentration of DMAMCL was determined in the plasma, liver, lung, kidney, and brain at different times (Fig. 2). The equations of calibration curve of DMAMCL were y = 0.012 x+0.0065 (n = 5) with r^2^ = 0.9972 (a correlation coefficients) in plasma, y = 0.0024 x+0.0172 (n = 5) with r^2^ = 0.9977 in brain, y = 0.00168 x + 0.0174 (n = 5) with r^2^ = 0.9968 in liver, y = 0.0058 x+0.158 (n = 5) with r^2^ = 0.9945 in kidney, y = 0.00646 x+0.028 (n = 5) with r^2^ = 0.9965 in spleen, y = 0.0034 x+0.0276 (n = 5) with r^2^ = 0.9982 in lung, and y = 0.00343 x+0.0341 (n = 5) with r^2^ = 0.9955 in heart, respectively. The recoveries in all tissues were ranged from 89.35% to 115.1%. The RSD of inter-day and intra-day was less than 10%. The results of biodistribution are shown in Fig. 2. The data revealed that DMAMCL accumulated and was dramatically higher in almost all tissues than in plasma. Importantly, in rat brain, DMAMCL was detectable and quickly reached a peak concentration of 18970 ± 9603 ng/ml at 0.5 hours after administration. More significantly, DMAMCL could still remain a higher concentration of 4913 ± 869 ng/ml for a longer period of time (3 hours) than in plasma (2566 ± 968 ng/ml). On the other hand, the fraction of C _brain_ / C _plasma_, an important index to evaluate drugs entering brain, represents the concentration ratio in brain and plasma[22, 23], can be used to evaluate the BBB penetration of drugs. Briefly, the larger the fraction is, the higher the concentration of DMAMCL accumulated in brain. The ratios of C _brain_ / C _plasma_ >1 represent a high ratio and indicate that a compound has a high BBB crossing. Therefore, we calculated the C _brain_ / C _plasma_ of DMAMCL. As shown in table 1, the relatively high ratios of C _brain_ / C _plasma_ revealed that DMAMCL could easily cross BBB and accumulate in the brain.

**Table 1:** Mean concentration of DMAMCL in plasma and brain at different time after administration.

	0.083h	0.5h	3h
Brain (ug/g)	6360	18970	4913
Plasma (ug/ml)	4373	10310	2567
C _brain_ / C _plasma_	1.45	1.84	1.91

### Anticancer activity of DMAMCL in vitro

**Fig 3:**
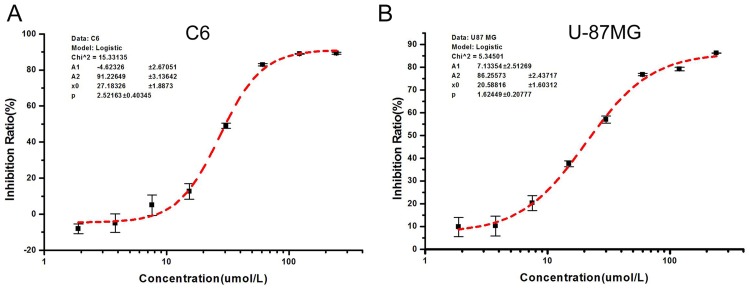
Inhibition of glioma cell growth by DMAMCL. C6 and U-87MG cells were treated with different concentrations of DMAMCL for 72 h. Cell viability was measured by MTT assays.

To determine the anticancer activity of DMAMCL in vitro, C6 and U-87MG cells were treated with or without DMAMCL for 72 h, and cell viability was measured by MTT assays. As shown in Fig. 3, DMAMCL inhibited the growth of both cell types, with IC_50_ values of 27.18 ± 1.89 μM for C6 cells and 20.58 ± 1.61 μM for U-87MG cells. DMAMCL inhibited growth of cultured C6 cells (Fig. 3A) and human U-87MG cells (Fig. 3B) in a concentration-dependent manner.

### DMAMCL induced apoptosis in C6 and U-87MG cells

**Fig 4:**
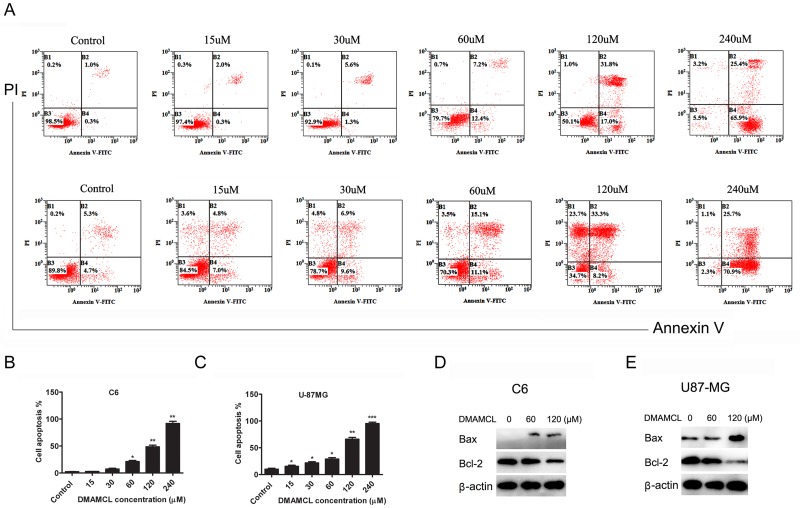
DMAMCL-induced apoptosis. C6 and U-87MG cells were treated with DMAMCL. After 24 h, cells were collected for Annexin V and PI detection or total protein extraction. (A) Representative FACS results. (B) and (C) The percentage of cell apoptosis (the total of early and late apoptosis) for C6 and U-87MG cells, respectively. (D) and (E) Western blotting detection for Bax, Bcl-2 and β-actin protein expression for C6 and U-87MG cells, respectively. One-way ANOVAs followed by Tukey’s post-hoc tests were used and statistical significance is indicated by asterisks (*p < 0.01 compared to vehicle; **p < 0.01 compared to vehicle; ***p < 0.001 compared to vehicle).

We analyzed the ability of DMAMCL to induce apoptosis in C6 and U-87MG cells using flow cytometry and Western blots (Fig. 4). Dual-labeled fluorescence activated cell sorting (FACS) analysis (Annexin V and PI) was used to measure early and late apoptosis and necrosis of C6 and U-87MG cells. Annexin V detects early apoptosis, whereas cells positive for PI and Annexin V are in late apoptosis. By 24 hr post-treatment, DMAMCL significantly increased early and late apoptosis and necrosis of C6 and U-87MG cells in a dose-dependent manner (Fig. 4 A, B and C). There were few surviving C6 or U-87MG cells after treatment with DMAMCL at 240 μM. The Bcl-2 family of regulator proteins influence apoptosis and have been widely studied in glioma cells and the development of MG [24]. Hence, we investigated whether DMAMCL could reduce anti-apoptotic protein Bcl-2 expression and induce pro- apoptotic protein Bax expression. Western blots showed that DMAMCL at 60 μM and 120μM up-regulated Bax expression and down-regulated Bcl-2 expression (Fig. 4 D and E).

### Efficacy of DMAMCL in a C6 rat tumor model

**Fig 5:**
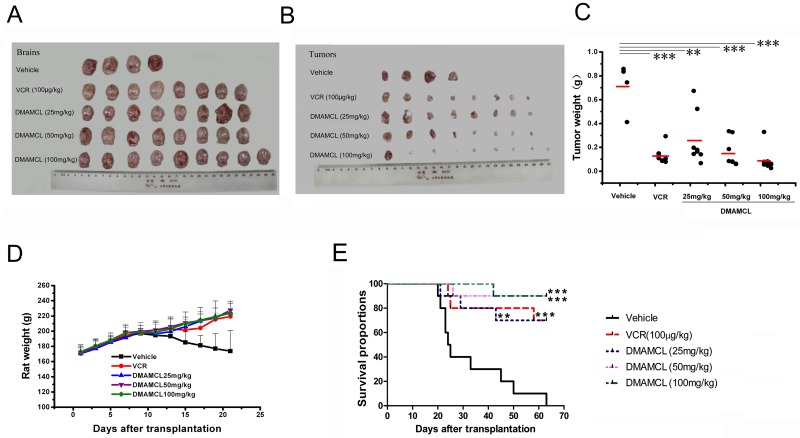
Treatment of intracerebral C6 glioma with DMAMCL. After euthanasia, brains and tumors were photographed, and tumors were weighed. Photographs of (A) whole brains and (B) tumors are shown. (D) Changes in rat body weight over time and (C) distributions of tumor weight for each group at the end of the experiment. Average tumor weight for each group is indicated by red lines. Statistical significance is indicated by asterisks (**p < 0.01 compared to vehicle; ***p < 0.001 compared to vehicle). E) Kaplan-Meier survival curves over 63 days. Rats were treated daily for 21 days with vehicle, VCR, or DMAMCL at 25, 50, or 100 mg/kg. (n = 10 rats/group). All groups were compared with the vehicle group. Significance is indicated by asterisks (**p < 0.01, ***p < 0.001).

To determine the anticancer activity of DMAMCL in vivo, intracerebral C6 tumors were established in Wistar rats and treated with DMAMCL. In the vehicle group, animals showed abnormal physical symptoms at early stages of disease. These symptoms included dramatic weight loss (Fig. 5D), loss of mobility, lethargy, hemorrhaging around the nose, eyes, and mouth, and hunched postures. Vehicle rats began dying on day 14, and a total of 5 rats died by the end of the experiment (survival rate = 44.4%). Additionally, many surviving animals developed hemiplegia.

Due to its serious neurotoxicity, VCR is commonly combined with other chemotherapeutic drugs in clinic [25]. In our study, VCR was used as the positive control. One rat died on day 18 in the VCR group, whereas 89% of rats treated with DMAMCL at 25 or 50 mg/kg survived to the end of the study (Table 2). The status of other animals was slump, but body weights increased significantly (Fig. 5D). Animals administered DMAMCL at 100 mg/kg displayed no signs of systemic or intracranial toxicity, gained weight normally (Fig. 5D), and 100% of animals in the group survived to the end of the study.

**Table 2:** DMAMCL inhibition of intracranial C6 tumor growth.

	Body weight (g)				
Group	Start	End	Tumor/brain (%)	Tumor weight (g)	Inhibition (%)
Vehicle	171.0 ± 9.5	173.7 ± 23.2	31.6 ± 10.7	0.71 ± 0.21	—
VCR	171.2 ± 5.6	219.3 ± 8.2**	6.5 ± 2.6***	0.13 ± 0.07***	81.6
DMA-25	170.5 ± 11.9	223.8 ± 13.1**	15.0 ± 9.5*	0.29 ± 0.20**	59.9
DMA-50	171.0 ± 7.9	227.5 ± 11.9***	6.3 ± 3.9***	0.15 ± 0.12***	78.7
DMA-100	173.0 ± 8.9	224.0 ± 12.1***	4.2 ± 3.1***	0.09 ± 0.09***	87.5

Note: DMA-25, DMA-50, and DMA-100 represent DMAMCL at doses of 25, 50, and 100 mg/kg, respectively. Inhibition represents the percent decrease in tumor weight compared to the vehicle group.

*p < 0.05,

**p < 0.01,

***p < 0.001 vs. vehicle.

Upon euthanasia, tumors were separated from brains and weighed. The weight distribution of tumors is shown in Fig. 5C. The average tumor weight of each group was: Vehicle, 0.71 ± 0.21 g; VCR, 0.13 ± 0.07 g; DMAMCL at 25 mg/kg, 0.29 ± 0.20 g; DMAMCL at 50 mg/kg, 0.15 ± 0.12 g; and DMAMCL at 100 mg/kg, 0.09 ± 0.09 g. Compared to the vehicle group, these weights corresponded to reductions in tumor weights by 81.6% for VCR, 59.9% for DMAMCL at 25 mg/kg, 78.7% for DMAMCL at 50 mg/kg, and 87.5% for DMAMCL at 100 mg/kg (Table 2). The highest dose of DMAMCL inhibited tumor growth more than VCR. The ratio of tumor weight: brain weight partly reflects the degree of tumor infiltration into the brain. Table 2 shows the ratios of tumor weight: brain weight after treatment with DMAMCL were less than those in the vehicle group (p < 0.001).

### Effects of DMAMCL on survival of C6 tumor-bearing rats

The survival of rats treated with DMAMCL was significantly greater than those in the vehicle group. Mean survival times for each group after implantation of C6 cells were: 31.8 ± 15.1 days for the vehicle group; 53.1 ± 16.8 days with DMAMCL at 25 mg/kg; 59.2 ± 12 days with DMAMCL at 50 mg/kg; and 60.8 ± 7 days with DMAMCL at 100 mg/kg. All rats in the vehicle group died, and tumor volumes ranged from 200 to 300 mm^3^ at 63 days after implantation. In contrast, DMAMCL-treated groups showed significantly improved survival at 63 days. The percentage of surviving rats was 70% after treatment with DMAMCL at 25 mg/kg, 90% after treatment with DMAMCL at 50 mg/kg, and 90% after treatment with DMAMCL at 100 mg/kg (Fig. 5E and Table 3). Even rats treated with the lowest dose of DMAMCL (25 mg/kg) had significantly greater survival than that in the vehicle group, and their survival rate was close to that of the VCR group.

**Table 3:** Effects of DMAMCL on C6 tumor-bearing rat survival.

	Survival (# of rats)		Time of death (d)					
Group	Start	End	Start	End	Survival (%)	Mean survival (d)	Median survival (d)	Median survival of deceased animals (d)
Vehicle	10	0	19	63	0	31.8±15.1	23.5	23.5
VCR	10	7	23	57	70***	54.5±16.4**	63	24
DMA-25	10	7	20	42	70**	53.1±16.8**	63	28
DMA-50	10	9	25	25	90***	59.2±12***	63	25
DMA-100	10	9	41	41	90***	60.8±7***	63	41

Note: DMA-25, DMA-50, and DMA-100 represent DMAMCL at doses of 25, 50, and 100 mg/kg, respectively.

*p < 0.05,

**p < 0.01,

***p < 0.001 versus vehicle.

### Histopathology

**Fig 6:**
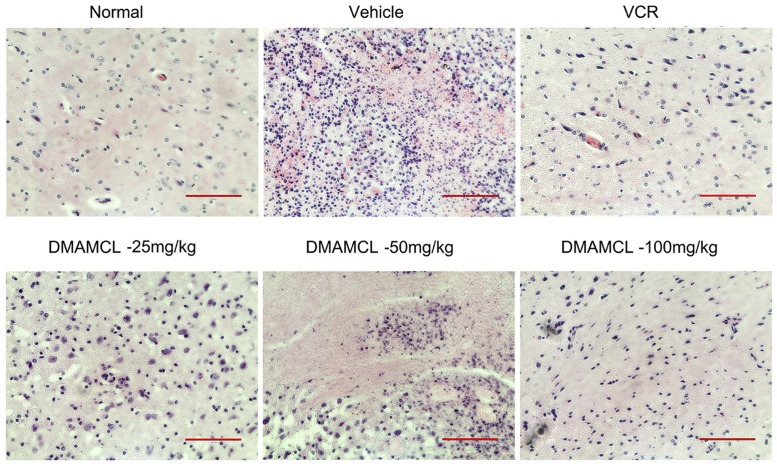
HE staining of brains from tumor-bearing rats after treatment for 21 days. All images are representative images, and scale bars indicate 100 μm.

The results of the histopathological analysis of animals treated with NS, VCR, or DMAMCL are shown in Fig. 6. The pictures are derived from ipsi lateral. The vehicle group exhibited substantial tumor cell in the brain tissue, sparse pink cytoplasm, densely stained round or oval vesicular nuclei, and atypical mitotic features. As the concentration of DMAMCL increased, the number of tumor cells in rat brain decreased. The brain tissues of animals treated with VCR or DMAMCL at 100 mg/kg appeared similar to normal brain tissues by histopathologic analysis.

### Toxicity of DMAMCL

**Fig 7:**
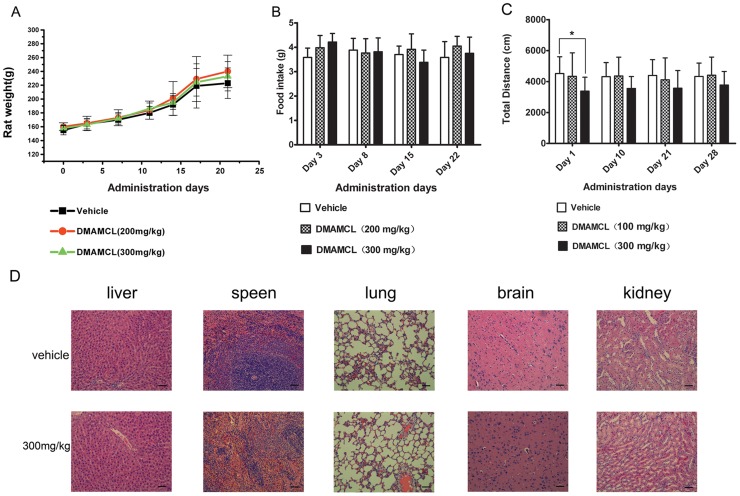
Toxicology evaluation of DMAMCL. A) and B) Effect of DMAMCL on body weight and food intake. DMAMCL (200 or 300 mg/kg) was orally administered once a day for 21 days and body weights were measured on days 0, 3, 7, 11, 14, 17, and 21 (n = 6 rats per group). Total food intake was measured as the weight of food consumed during a 24-h period on days 3, 8, 15, and 22. C) Locomotor activities of rats Rats were orally administered with or without DMAMCL (either 100 or 300 mg/kg) once a day for 21 days. The individual total distance of rats was monitored during a test period of 60 min on days 1, 10, 21 and 28. Statistical significance is indicated by asterisks (*p < 0.05 compared to vehicle). D) HE staining of rat tissues after daily treatment with vehicle or DMAMCL at 300 mg/kg for 21 days. Representative HE staining results are shown. Scale bars indicate 100μm.

The toxicity of DMAMCL was evaluated in rats after oral administration of 100, 200 or 300 mg/kg DMAMCL once a day for 21 days. Body weight, daily food intake, histopathology, locomotor activities and hematological and serum biochemical measurements were evaluated. No significant changes were observed in rat body weight or mean daily food intake (Fig. 7A and B), histopathology (Fig. 7D), and hematology (Table 4) or serum biochemical parameters (Table 5) following treatment with DMAMCL at either dose. In order to assess neurological effects of DMAMCL, locomotor activity was performed. The results indicated that at the first day the total distance in rats treated with DMAMCL-300mg/kg decreased compared with vehicle group (Fig. 7C). In the following days, rats in DMAMCL-300mg/kg group were still less active than vehicle group, but without significant statistical difference in the total distance (Fig. 7C). And no significant changes were observed in the total distance of DMAMCL-300mg/kg group within one week of DMAMCL withdrawal (Fig. 7C). Rats in DMAMCL-100mg/kg group were as active as vehicle group during the observation period.

**Table 4:** Effects of DMAMCL on hematological parameters.

Parameter	Vehicle	DMAMCL(200 mg/kg)	DMAMCL(300 mg/kg)
RBC (×10^12^/L)	6.9 ± 1.5	6.73±1.3	6.37 ± 1.01
MCV (fL)	57.1 ± 1.0	55.5 ± 1.4	53.5 ± 2.3
RDW%	18.1 ± 0.5	18.5 ± 1.1	17.8 ± 0.7
HCT%	37.2 ± 1.0	35.6 ± 1.2	37.7 ± 0.8
PLT (×10^9^/L)	1244 ± 105	1195 ± 87	1132 ± 82
WBC (×10^9^/L)	13.3 ± 3.4	12.5 ± 2.5	11.9 ± 1.3
HGB (g/L)	135 ± 15.1	145 ± 12.3	140 ± 11.1
LYM%	67.5 ± 5.8	71.3 ± 6.3	69.9 ± 6.0
GRAN%	29.1 ± 3.2	26.5 ± 3.3	26.3 ± 4.1
MONO%	3.5 ± 1.1	2.1 ± 0.3	2.9 ± 0.9

Abbreviations: RBC, red blood cell count; MCV, mean corpuscular volume; RDW, mean width of erythrocyte distribution; HCT, hematocrit; PLT, platelet count; WBC, white blood cell count; HGB, hemoglobin; LYM%, percent of lymphocytes; GRAN%, percent of granulocytes; and MONO%, percent of monocytes.

**Table 5:** Effects of DMAMCL on the serum biochemistry of rats.

Parameter	Vehicle	DMAMCL(200 mg/kg)	DMAMCL(300 mg/kg)
ALT (U/L)	52 ± 9	51 ± 10	49 ± 11
AST (U/L)	103 ± 10	98 ± 12	111 ± 15
GLU (mM)	4.98 ± 1.54	5.22 ± 2.01	5.23 ± 1.34
Urea (mM)	7.01 ± 1.12	6.45 ± 2.3	7.33 ± 1.02
Cr (mM)	85 ± 15	79 ± 23	80 ± 17
TP (g/L)	57.5 ± 5.5	61.0 ± 4.6	58.3 ± 3.3
ALB (g/L)	31 ± 2.33	32.5 ± 3.01	34 ± 1.98
CHOL (mM)	1.45 ± 0.35	1.39 ± 0.67	1.42 ± 0.78
TG (mM)	0.77 ± 0.24	0.81 ± 0.23	0.69 ± 0.33
CK (U/L)	405 ± 98	423 ± 35	523 ± 101
ALP (U/L)	182 ± 22	190 ± 19	179 ± 30

Abbreviations: ALT, alanine aminotransferase; AST, aspartate aminotransferase; GLU, glucose; BUN, blood urea nitrogen; Cr, creatinine; TP, total protein; ALB, albumin; CHOL, cholesterol; TG, triglycerides; CK, creatine kinase; and ALP, alkaline phosphatase.

## Discussion

In this present study, we evaluated the anti-cancer effect of DMAMCL, an MCL derivative, on C6 and U-87MG cells. Much research supports a remarkable activity for DMAMCL to glioma, including biodistribution of DMAMCL, potential to inhibit the growth of glioma cells, and probable mechanisms of targeting Bcl-2 signal pathway. Briefly, we successfully generated a rat model of glioma and assessed the anticancer activity of DMAMCL. We found that DMAMCL could efficiently inhibit the growth of C6 and U-87MG cells. Most importantly, we found a mechanism for the anticancer activity of DMAMCL in vitro: DMAMCL may target Bcl-2 signal pathway to attack tumor cells.

In order to investigate the anti-cancer activity of DMAMCL, the first step is to establish a well glioma mode, since rat models of intracranial brain tumors are essential in the neuro-oncology field. Our intracranial glioma model was established by directly injecting a mixture of glioma cells and matrigel into the caudatum. Compared with other commonly adopted models, where a cell suspension or tumor fragment [26, 27] is injected directly into the caudatum, the use of matrigel prevents the cell suspension from refluxing and minimizes the development of extracranial tumors. Additionally, this unassisted or “hands-free” approach to intracranial tumor implantation not only improves tumor growth but also reduces procedure time and mortality.

It is well known that an intense resistance to death-inducing stimuli is the biological hallmark of MG. Currently, multiple biology measurements have been performed to research anti-glioma drugs. Honokiol, a natural compound, was reported to reduce the volumes of rat 9L gliosarcomas by 52.77% and human U251 glioma xenografts by 50.21%, but merely increased survival by 5 days compared to controls [12]. Rapamycin inhibits mTOR. It is clinically used as an immunosuppressant after solid-organ transplantation and as an antitumor agent in renal and breast cancers [28, 29]. Tyler et al. [26] reported that direct injection of rapamycin into the brain using biodegradable beads increased survival from 12.5 to 29 days in a rat 9L gliosarcoma model. However, direct injection into gliomas is clinically limited. Dan Li et al. [27] designed a novel CLA-PTX microemulsion which showed a significant anti-glioma efficacy by self-emulsifying to form nanosized microemulsion droplets. But this technology also encountered the risk of the encapsulation efficiency declining due to the change of microemulsion physical stability. Therefore, MG therapies that are safe, efficient, easy to deliver, and affordable are desperately needed. In our experiments, the pharmacodynamic evaluation of DMAMCL, which is easy to manipulate and cost-effective, was performed. The results in vitro and in vivo demonstrate that DMAMCL could effectively inhibit the growth of, and induce apoptosis in, C6 or human U-87MG cells.

Firstly, in this study, the biodistribution of DMAMCL revealed greater concentrations of MCL in the brain. As is known, drugs must cross the BBB in vivo to exert a therapeutic effect. For many therapies, the BBB represents a formidable obstacle. The slow metabolism of DMAMCL to MCL allowed sustained release of MCL over time and the accumulation of MCL indicated that DMAMCL can cross the BBB. All of these indicate that DMAMCL might be a potentially valuable and safe brain tumor therapy.

Additionally, in vitro Western blots indicated a remarkable reduction of Bcl-2 and increase of Bax following DMAMCL treatment. The Bcl-2 family of regulator proteins controls apoptosis and influences the permeability of the outer mitochondrial membrane. The family includes pro- (Bax, Bad, Bak, and Bok) and anti-apoptotic factors (Bcl-2, Bcl-xl, and Bcl-w). Previous studies showed that PTL targeted NF-kB, Stat3, Bcl-2, reactive oxidative species (ROS) in cancer cells [30–33]. And Bcl-2 proteins were widely studied in glioma cells and the development of MG [24, 34]. In our study, the Bcl-2 family genes were preliminarily analyzed to determine whether DMAMCL target this signal pathway in glioma cells. Undoubtedly, a better understanding of drug mechanisms may lead to better therapies for primary brain tumors. Therefore, future studies are expected to focus specifically on investigation of DMAMCL mechanisms.

Most importantly, our in vivo results from C6 intracranial brain tumor models indicated that DMAMCL could efficiently inhibit the growth of C6 gliomas in rats. Dramatic dose-dependent efficacy was observed, with a maximal inhibitory rate of 87.5%. Especially, the tumor: brain weight ratio was significantly reduced by DMAMCL treatment, indicating the degree of tumor being inhibited. In contrast, control rats lost weight, began to die on 14th day, had a survival rate of only 50%, and many animals developed hemiplegia. Nevertheless, tumor-bearing rats treated with DMAMCL at 100 mg/kg exhibited no signs of systemic or intracranial toxicity and gained weight normally. On the other hand, oral administration of DMAMCL at 200 or 300 mg/kg once a day for 21 days did not lead to toxicity. Body weight, daily food intake, histopathology, hematology, locomotor activity and serum biochemistry were normal. Most significantly, DMAMCL-treated rats survived ~30 days longer than vehicle animals. Resent data suggest that MG patients survive < 6 months without treatment. Even after intensive therapy, including gross resection, radiation, and chemotherapy, the mean survival time is only 14 months [13]. Cisplatin combined with thalidomide increased the survival time from 18 days to 20.5 days in a 9L rat gliosarcoma model [35], and Honokiol only increased survival by 5 days [12]. Therefore, the prolongation of survival time for this aggressive tumor might be promising in the life extension for a glioma patient.

Taken together, both in vitro and in vivo results suggest that DMAMCL effectively inhibited the glioma cell growth without systemic and local toxicity. DMAMCL crossed the BBB and contributed to the anti-glioma activity. Therefore, DMAMCL might be a potential therapeutic for MG treatment. Further investigations of DMAMCL interactions with the tumor environment are supposed to result in illuminations on the therapeutic mechanisms of DMAMCL as well as bring about novel MG therapies.

## References

[1] Mrugala MM (2013) Advances and challenges in the treatment of glioblastoma: a clinician’s perspective. Discov Med 15: 221–230. 23636139

[2] Westphal M, Lamszus K (2011) The neurobiology of gliomas: from cell biology to the development of therapeutic approaches. Nat Rev Neurosci 12: 495–508. 10.1038/nrn3060 21811295

[3] Sengupta S, Marrinan J, Frishman C, Sampath P (2012) Impact of temozolomide on immune response during malignant glioma chemotherapy. Clin Dev Immunol 2012: 831090 10.1155/2012/831090 23133490 PMC3486128

[4] DeAngelis LM (2001) Brain tumors. N Engl J Med 344: 114–123. 11150363 10.1056/NEJM200101113440207

[5] Stupp R, Mason WP, van den Bent MJ, Weller M, Fisher B, et al (2005) Radiotherapy plus concomitant and adjuvant temozolomide for glioblastoma. N Engl J Med 352: 987–996. 15758009 10.1056/NEJMoa043330

[6] Lefranc F, Brotchi J, Kiss R (2005) Possible future issues in the treatment of glioblastomas: special emphasis on cell migration and the resistance of migrating glioblastoma cells to apoptosis. J Clin Oncol 23: 2411–2422. 15800333 10.1200/JCO.2005.03.089

[7] Stupp R, Hegi ME, Mason WP, van den Bent MJ, Taphoorn MJ, et al (2009) Effects of radiotherapy with concomitant and adjuvant temozolomide versus radiotherapy alone on survival in glioblastoma in a randomised phase III study: 5-year analysis of the EORTC-NCIC trial. Lancet Oncol 10: 459–466. 10.1016/S1470-2045(09)70025-7 19269895

[8] Brain and Spinal Cord Tumors in Children (2010) American Cancer Society Atlanta, Ga, USA.

[9] National Cancer Institute (2010) FDA Approval for Temozolomide. Available: www.cancer.gov/cancertopics/druginfo/fda-temozolomide.

[10] Sampson JH, Aldape KD, Archer GE, Coan A, Desjardins A, et al (2011) Greater chemotherapy-induced lymphopenia enhances tumor-specific immune responses that eliminate EGFRvIII-expressing tumor cells in patients with glioblastoma. Neuro Oncol 13: 324–333. 10.1093/neuonc/noq157 21149254 PMC3064599

[11] Newlands ES, Stevens MF, Wedge SR, Wheelhouse RT, Brock C (1997) Temozolomide: a review of its discovery, chemical properties, pre-clinical development and clinical trials. Cancer Treat Rev 23: 35–61. 9189180 10.1016/s0305-7372(97)90019-0

[12] Wang X, Duan X, Yang G, Zhang X, Deng L, et al (2011) Honokiol crosses BBB and BCSFB, and inhibits brain tumor growth in rat 9L intracerebral gliosarcoma model and human U251 xenograft glioma model. PLoS One 6: e18490 10.1371/journal.pone.0018490 21559510 PMC3084695

[13] Nieto-Sampedro M, Valle-Argos B, Gómez-Nicola D, Fernández-Mayoralas A, Nieto-Díaz M (2011) Inhibitors of glioma growth that reveal the tumour to the immune system. Clin Med Insights Oncol 5:265–314. 10.4137/CMO.S7685 22084619 PMC3201112

[14] Anderson KN, Bejcek BE (2008) Parthenolide induces apoptosis in glioblastomas without affecting NF-kappaB. J Pharmacol Sci 106: 318–320. 18277052 10.1254/jphs.sc0060164

[15] Jin P, Madieh S, Augsburger LL (2007) The solution and solid state stability and excipient compatibility of parthenolide in feverfew. AAPS PharmSciTech 8: E105 10.1208/pt0804105 18181526 PMC2750691

[16] Zhang Q, Lu Y, Ding Y, Zhai J, Ji Q, et al (2012) Guaianolide sesquiterpene lactones, a source to discover agents that selectively inhibit acute myelogenous leukemia stem and progenitor cells. J Med Chem 55: 8757–8769. 10.1021/jm301064b 22985027

[17] Kilkenny C, Browne WJ, Cuthill IC, Emerson M, Altman DG (2010) Improving bioscience research reporting: the ARRIVE guidelines for reporting animal research. PLoS Biol 8: e1000412 10.1371/journal.pbio.1000412 20613859 PMC2893951

[18] Zhai JD, Li D, Long J, Zhang HL, Lin JP, et al (2012) Biomimetic semisynthesis of arglabin from parthenolide. J Org Chem 77: 7103–7107. 10.1021/jo300888s 22849854

[19] Chen Y, Zhang Q, Zhai JD, Ma WW, Fan HX, Zhang F (2010) W. C. N. Patent 201,010,153,701.0.

[20] von Eckardstein KL1, Patt S, Kratzel C, Kiwit JC, Reszka R (2005) Local chemotherapy of F98 rat glioblastoma with paclitaxel and carboplatin embedded in liquid crystalline cubic phases. J Neurooncol 72: 209–215. 15937642 10.1007/s11060-004-3010-6

[21] Guo M, Roman RJ, Fenstermacher JD, Brown SL, Falck JR, et al (2006) 9L gliosarcoma cell proliferation and tumor growth in rats are suppressed by N-hydroxy-N’-(4-butyl-2-methylphenol) formamidine (HET0016), a selective inhibitor of CYP4A. J Pharmacol Exp Ther 317: 97–108. 16352703 10.1124/jpet.105.097782

[22] Feng MR (2002) Assessment of blood-brain barrier penetration: in silico, in vitro and in vivo. Curr Drug Metab 3: 647–657. 12369891 10.2174/1389200023337063

[23] Abbott NJ (2004) Prediction of blood-brain barrier permeation in drug discovery from in vivo, in vitro and in silico models. Drug Discov Today Technol 1:407–416. 10.1016/j.ddtec.2004.11.014 24981621

[24] Nagane M, Levitzki A, Gazit A, Cavenee WK, Huang HJ (1998) Drug resistance of human glioblastoma cells conferred by a tumor-specific mutant epidermal growth factor receptor through modulation of Bcl-XL and caspase-3-like proteases. Proc Natl Acad Sci U S A 95:5724–5729. 9576951 10.1073/pnas.95.10.5724 PMC20446

[25] Colpo A, Wilson FH, Nardi V, Hochberg E (2012) Administration of vincristine in a patient with Machado-Joseph disease. Oncology 82:165–167. 10.1159/000336602 22433430 PMC3701890

[26] Tyler B, Wadsworth S, Recinos V, Mehta V, Vellimana A, et al (2011) Local delivery of rapamycin: a toxicity and efficacy study in an experimental malignant glioma model in rats. Neuro Oncol 13: 700–709. 10.1093/neuonc/nor050 21727209 PMC3129273

[27] Li D, Yang K, Li JS, Ke XY, Duan Y, et al (2012) Antitumor efficacy of a novel CLA-PTX microemulsion against brain tumors: in vitro and in vivo findings. Int J Nanomedicine 7: 6105–6114. 10.2147/IJN.S38927 23269869 PMC3529648

[28] Hudes G, Carducci M, Tomczak P, Dutcher J, Figlin R, et al (2007) Temsirolimus, interferon alfa, or both for advanced renal-cell carcinoma. N Engl J Med 356: 2271–2281. 17538086 10.1056/NEJMoa066838

[29] Chan S, Scheulen ME, Johnston S, Mross K, Cardoso F, et al (2005) Phase II study of temsirolimus (CCI-779), a novel inhibitor of mTOR, in heavily pretreated patients with locally advanced or metastatic breast cancer. J Clin Oncol 23: 5314–5322. 15955899 10.1200/JCO.2005.66.130

[30] Dell’Agli M, Galli GV, Bosisio E, D’Ambrosio M (2009) Inhibition of NF-kB and metalloproteinase-9 expression and secretion by parthenolide derivatives. Bioorg Med Chem Lett 19: 1858–1860. 10.1016/j.bmcl.2009.02.080 19269818

[31] Guzman ML, Rossi RM, Neelakantan S, Li X, Corbett CA, et al (2007) An orally bioavailable parthenolide analog selectively eradicates acute myelogenous leukemia stem and progenitor cells. Blood 110: 4427–4435. 17804695 10.1182/blood-2007-05-090621 PMC2234793

[32] Carlisi D, D’Anneo A, Angileri L, Lauricella M, Emanuele S, et al (2011) Parthenolide sensitizes hepatocellular carcinoma cells to TRAIL by inducing the expression of death receptors through inhibition of STAT3 activation. J Cell Physiol 226:1632–1641. 10.1002/jcp.22494 21413021

[33] Sun J, Zhang C, Bao YL, Wu Y, Chen ZL, et al (2014) Parthenolide-induced apoptosis, autophagy and suppression of proliferation in HepG2 cells. Asian Pac J Cancer Prev 15: 4897–4902. 24998560 10.7314/apjcp.2014.15.12.4897

[34] Strik H, Deininger M, Streffer J, Grote E, Wickboldt J, et al (1999) BCL-2 family protein expression in initial and recurrent glioblastomas: modulation by radiochemotherapy. J Neurol Neurosurg Psychiatry 67: 763–768. 10567494 10.1136/jnnp.67.6.763 PMC1736652

[35] Murphy S, Davey RA, Gu XQ, Haywood MC, McCann LA, et al (2007) Enhancement of cisplatin efficacy by thalidomide in a 9L rat gliosarcoma model. J Neurooncol 85: 181–189. 17534579 10.1007/s11060-007-9406-3

